# Can Heart Rate Variability (HRV) Be Used as a Biomarker of Thermal Comfort for Mine Workers?

**DOI:** 10.3390/ijerph18147615

**Published:** 2021-07-17

**Authors:** Guoshan Wu, Heqing Liu, Shixian Wu, Guanglei Liu, Caihang Liang

**Affiliations:** 1 School of Resource & Environment and Safety Engineering, Hunan University of Science and Technology, Xiangtan 411201, China; wushx@guat.edu.cn (S.W.); 190101030003@mail.hnust.edu.cn (G.L.); 2 School of Energy and Building Environment Engineering, Guilin University of Aerospace Technology, Guilin 541004, China; 3 School of Mechanical and Electrical Engineering, Guilin University of Electronic Technology, Guilin 541004, China; lianghang@guet.edu.cn

**Keywords:** heart rate variability, thermal comfort, mining environment, working body

## Abstract

This study aimed to determine whether heart rate variability (HRV) can express the thermal comfort of mine workers. Eight subjects ran on a treadmill (5.5 km/h) to simulate heavy labor in three kinds of mining environments (22 °C/90%, 26 °C/90%, 30 °C/90%), respectively. Based on the measured electrocardiogram (ECG) data, the HRV of the subjects was calculated. The results showed that the HRV indices changed obviously under different temperature environments. In the neutral and hot environment, except for the LF, TP and LF/HF, there were significant differences in each index. However, there was no significant difference between the cold and neutral environments. The R-R intervals, the very low-frequency power (VLF), pNN20 and SampEN had strong negative correlation with the thermal sensation of people from sitting to work (ρ < −0.700). These indices may be used as thermal comfort predictive biomarkers of mine workers.

## 1. Introduction

Coal is an important energy source for various countries [1]. With the reduction in shallow resources and the continuous demand for minerals, mines have become much deeper, and the temperature and humidity of mining workfaces have become much higher [2,3,4]. In deep mining, workers are engaged in heavy physical labor, they are often in a state of thermal discomfort [5]. The combined effect of high temperature and high humidity may put workers’ health at risk [6,7]. Moreover their dissatisfaction with these working conditions is growing [7,8,9]. In addition, the body sweats a lot after heavy labor, and the clothes are often wet. The workers may feel cold and uncomfortable in the mine ventilation. Therefore, we should pay more attention to the thermal comfort of mine workers. At present, direct indices, empirical indices and rational indices have been used in thermal comfort research and evaluation of mining environments. In the early days, people mainly used the combination of single or multiple climate parameters, such as ambient temperature and black ball temperature, to evaluate the thermal comfort of the underground environment. In many countries, such as the United States, the United Kingdom and Germany, the effective temperature (ET) has been used to evaluate the underground environment, and the labor management system has been formulated based on it [10]. WBGT was reliable and easy to use, and it can predict thermal comfort zone more accurately than the DI index [3]. WBGT has become the most commonly used index in underground mines [9]. PMV/PPD is the international thermal comfort index, but it is not suitable for active humans with a high metabolic rate. The PHS model is a universal thermal stress model [11]. It has been widely recognized in the world. There was no significant difference between the predicted values of the PHS model and measured values of human core temperature in underground mines [7]. Many mining enterprises use it to conduct thermal stress assessment management [12,13,14,15] and study the movable refuge chambers of mining [14]. However, the PHS model focuses only on human thermal stress, not on psychology. The metabolic rate and clothing thermal resistance of the UTCI are fixed [16]. Therefore, it is not suitable for mines. Numerous thermal comfort indices have their own advantages, but none of them is suitable for all environmental conditions [16]. If the selected index is unsuitable, it will lead to inaccurate identification of human thermal discomfort in mines.

Thermal comfort is a subjective assessment of personal psychological satisfaction with the thermal environment [17]. Human physiology, psychology and personal psychophysics are the precursors of human thermal comfort [18]. Under the stimulation of the external environment, the human body strives to maintain homeostasis [19]. When exposed to a cold environment, the activity of the sympathetic nerve of the hypothalamus causes vasoconstriction [20], the skin blood flow is reduced, and the heat dissipation of the skin is decreased. In addition, the activity of the sympathetic nerve induced by a cold environment may also increase heat production, further maintaining the body heat balance. A hot environment can cause the sympathetic nerve to be activated, and the sympathetic nerve of the cerebral cortex is dominant [20]. Sweat glands were activated and accompanied by skin vasodilation, and the heat dissipation of skin is enhanced. When people are in a neutral environment, the parasympathetic nerve is active, and the sympathetic nerve is inhibited. Therefore, human thermal comfort may be essentially regulated by the autonomic nervous system composed of sympathetic and parasympathetic nerves [21]. HRV describes the changes between successive heartbeats [22]. It can be used to evaluate the tension and balance of the myocardial sympathetic nerve between accessory nerves indirectly and quantitatively [23]. The R-R interval and the coefficient of variation in R-R intervals may be considered worthy of further study as objective indications of the effect of the external environment on people [24]. The minimum values of LF and the LF/HF appeared in a neutral thermal environment, and these indices increased with the ambient temperature [25]. The LF/HF of the human body in an uncomfortable environment was significantly higher than that in a comfortable environment [26]. The sympathetic nerve of outdoor workers is more active in winter than in summer [27]. The higher the LF/HF is, the stronger the thermal discomfort [21]. In addition, some studies have suggested that the VLF was associated with the thermoregulation [28]. It can better reflect the effect of the nervous system on the heart rate variability than other HRV indices, and it may be a good index of thermal sensation [18]. The SampEn was an important method to evaluate the immeasurability of the ECG signal. It was also considered very important to the thermal state of subjects in some studies [18].

Previous studies have shown that HRV is related to the thermal comfort of the human body. Some studies have shown that some indicators of the HRV can be used as biomarkers of human thermal comfort. However, their research were more based on sitting or resting people. In the mining workface, the air humidity is often close to saturation, and the workers are engaged in high-intensity labor in cotton long sleeve overalls. Compared with the surface or building environment, there are great differences in environmental parameters, clothing and metabolic rate of the human body. Therefore, it is of great significance to study the relationship between heart rate variability and thermal sensation in miners. In this paper, the environments associated with mining workfaces were simulated in an artificial environment cabin, and 8 subjects were employed to run on the treadmill (5.5 km/h) to simulate heavy labor. ECG data of the subjects during the experiment were recorded with a dynamic ECG recorder, and the HRV changes of the subjects were studies. The relationship between the HRV indices and the subjective thermal sensation was investigated through a questionnaire survey.

## 2. Materials and Methods

### 2.1. Participants and Clothing

Eight male college students (an average age of 21.1 ± 0.6 years, an average height of 167.7 ± 4.9 cm, and an average weight of 63.7 ± 8.5 kg) with good health and no history of heat intolerance or respiratory or vascular diseases were recruited to participate in the study. Before the experiment, the subjects were informed about the requirements, objectives, procedure, potential risks and possible physical discomfort of the test. During the experiment, the subjects could choose to quit the test at any time. Subsequently, the two sides signed an informed consent form approved by the Ethics Committee of Guilin University of Electronics and Technology. Before the test, the subjects were forbidden to drink alcohol for 24 h, and were required not to drink tea or coffee, smoke or perform heavy exercise for at least 2 h before each test.

To make the experimental effect closer to the actual situation of the mines, the subjects were wearing shorts underneath mining overalls. The overalls, namely, a long sleeve top and trousers, were made of pure cotton with a thermal resistance of 0.164 (m^2^·K)/W and wet resistance of 36.12 (Pa·m^2^)/W.

### 2.2. Experimental Procedure

The preparation process was conducted in a room with an air temperature of 26 °C. ECG recorders (Healink Ltd., Bengbu, China) were mounted at designated sites on the subjects and then coveralls were donned. The entire preparatory process lasted approximately 15 min. When the temperature and humidity of the cabin reached the experimental requirements, the ECG recorder was turned on, and the subjects entered the environmental chamber and sat still for 25 min. They came out of the cabin and entered the preparation room to rest for 5 min. Water was allowed during rest. The subjects entered the cabin and began the first-stage running test with a speed controlled at 5.5 km/h. Running at this speed is equivalent to heavy labor [11]. After running for 25 min, the subjects went to the preparation room and rested for 5 min. Running and resting were repeated 2 more times, and the whole experiment was completed. The procedure of the test is shown in Table 1. The study was conducted in accordance with the Declaration of Helsinki, and the protocol was approved by the Ethics Committee of Guilin University of Electronics and Technology.

### 2.3. Test Conditions

An artificial environmental chamber was used to simulate the environment of the underground workface, and its dimensions were 3 m × 2.5 m × 2.2 m (length × width × height). The artificial environment chamber consisted of a refrigeration unit, electrical heating equipment, vapor humidification unit, fan, data collection unit, and computer. The environmental parameter control system provided control over the temperature of the air in the compartment, humidity, wind speed, and wall temperature. The dry bulb temperature of the cabin could be controlled in the range of −15 °C to 50 °C (±0.5 °C), and the relative humidity could be controlled in the range of 30–95% (±2%). The temperature of the cabin was calibrated before the test using a primary standard thermometer (minimum 0.05 °C, Hongxing Instrument Factory, Hengshui, China) and the humidity was calibrated using a JT-IAQ indoor thermal environment comfort tester (precision ±1.5%, Beijing Century Jiantong Technology Co., Ltd., Beijing, China). In some deep mines, the air temperature in the workface may exceed 26 °C. In high temperature mines the air temperature reaches 35 ℃, and the relative humidity often exceeds 85% [2,4]. To protect the workers and ensure production safety, as specified in the Safety Code for Coal Mines of China, the working times must be shortened when the air temperature of the workface exceeds 26 °C, and the operation must be stopped when the air temperature of the workface exceeds 30 ℃. Therefore, the environmental parameters in this test were controlled at 22 °C/90%, 26 °C/90% and 30 °C/90%, which represent three thermal conditions: cold, neutral and hot, respectively. The airspeed was controlled at 1.5 m/s, and the wall radiation temperature was equal to the air temperature, as shown in Table 2. The temperature conditions in the test process were random, and they were not carried out in the order of temperature increasing step by step.

### 2.4. Test Scenarios

**Figure 1:**
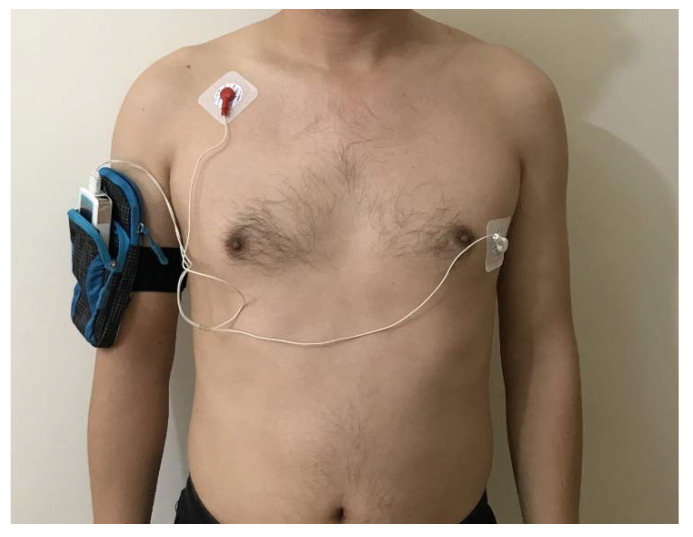
The Holter device and the connection method.

The HRV was calculated based on the ECG data measured by the Holter device (Healink-R211, bandwidth 0.05–40 Hz, Bengbu, China). The HRV is the change between consecutive R-R intervals, so it is essential to acquire the R-wave signal during the measurement. R-wave signals can usually be obtained in the V5 and V6 leads in the chest [29]. In this test, two electrodes (Shanghai Junkang Medical Equipment Co., Ltd., Shanghai, China) were used for measurements. The V5 lead was used to connect to the negative electrode (white), which was positioned at the intersection of the fifth ribs and the central line of the left clavicle, close to the left ventricle. In addition, the monopolar limb lead RA was chosen for the measurement point 10 cm below the center of the placed right clavicle. This was done to remove interference of arm movement on the ECG signal [30]. The leading RA was connected to the positive electrode (red). The sampling rate of the device was set to 100 Hz, 250 Hz or 400 Hz, for a more precise adoption rate, the sampling rate of the electrocardiographic recorder was set to 400 Hz [31]. The Holter device used in the test and its connection are shown in Figure 1. To obtain the HRV of the subjects under each environmental condition, the time series data of the R-R interval were extracted by ECG viewer 1.2 software (Healink Ltd., Bengbu, China).

### 2.5. Questionnaire Survey

A questionnaire was administered simultaneously during the experiment to assess the subjects’ thermal sensation and thermal comfort status. The questionnaires were distributed to the subjects at the beginning of the test, and the subjects were asked to fill out a thermal sensation vote in the last 1 min of the preparatory stage, and at 15 and 25 min of each stage of sitting and running. Considering that the metabolic rate of the subject in this test was relatively large, a new thermal sensation scale was obtained by modifying the thermal sensation scale proposed by the American Society of Heating, Refrigeration and Air Conditioning Engineers. The new thermal sensation scale contains 9 thermal sensation levels, which are extremely cold (−4), very cold (−3), cold (−2), slightly cold (−1), neutral (0), warm (1), hot (2), very hot (3), and extremely hot (4), respectively.

### 2.6. HRV Calculation

The HRV is usually analyzed by the time-domain method, spectral-domain method and nonlinear method. In this study, three methods were utilized to calculate the HRV indices summarized in Table 3. The time-domain HRV indices are easy to compute and simple. They can describe the beat-to-beat variability by using statistical techniques. In this group, the R-R interval and SDNN are the basic parameters of HRV. The R-R interval may be considered worthy of further study as an objective indication of the effect on people of the external environment [24]. It has relatively distinct discrimination between different cold and hot environments [18]. The SDNN is the standard deviation of the NN interval, which is the simplest variable of the HRV. The RMSSD represents the square root of the mean squared differences between successive RR intervals. The pNN20 represents the percentage of the RR consecutive pairs that differ by 20 milliseconds. The RMSSD and pNN20 are the most commonly used measures of heart rate interval difference, which estimate the high-frequency changes of heart rate, so they are highly correlated. Unlike the time-domain method, the spectral-domain method provides a greater understanding of heartbeat variation by decomposing the ECG into fundamental frequency components. Five indices of the frequency domain analysis method were selected, namely, the LF, HF, VLF, total power (TP) and LF/HF. The LF (0.04–0.15 Hz) changes with the change of thermal environment, [25,32] the HF (0.15–0.4 Hz) is generally considered to be the origin of the vagus nerve. The LF/HF, the ratio of low frequency component to the high frequency component, reflects the balance between sympathetic and parasympathetic nerves. It also considers to be related to human thermal comfort [21,25,26,27]. The VLF refers to the power in the frequency band below 0.04 Hz, which is related to human body temperature regulation. The SampEN is the probability that two sequences match if a new sample is added to the sequence. The VLF and SampEN were considered a good index of human thermal sensation [18]. Therefore, it is feasible to use these indices to study the relationship between workers and the thermal comfort associated with to underground mining environment.

ECG data were recorded continuously during the experiment. For each environmental condition, the human body needs to adapt to changes in the thermal environment for a certain time. During the stages of sitting or running, ECG data from the 10th–15th and the 20th–25th min were extracted for HRV analysis. After the data acquisition, the ECG was imported into the ECG viewer software for visual inspection to confirm that the recorded waveform has relatively high signal quality. Based on the QRS detection module, R wave can be automatically detected to reduce noise, baseline drift and other components, highlight peak and smooth near the peak. The second round of visual examination was performed to remove the misidentified peaks and ectopic pulsations. We confirmed that no ectopic pulsation occurred in these subjects. Finally, the continuous RR interval data are more than 256. HRV indices were calculated by Kubios Hrv3.3, a professional software developed by the University of Kuobios, Kuopio, Finland. An advanced detrended method was included in Kubios HRV software to remove the non-stationarity of the RR interval time series. This method works like a time-varying high pass filter. The data was smoothed according to the value of smoothing parameters. The bigger the smoothing parameter is, the lower the cut-off frequency of the filter is. In this process, the cut-off frequency was below the low frequency band (<0.04 Hz), ensuring that no part of the normal short-term HRV was removed. The Fourier transform (FFT) method was employed to calculate the power spectral density (PSD) of the R-R interval sequence in the spectrum domain analysis. The spectrum bands of analysis included VLF ranging from 0.00 to 0.04 Hz, low-frequency power (LF) ranging from 0.04 to 0.15 Hz, and high-frequency power (HF) ranging from 0.15 to 0.4 Hz.

### 2.7. Statistical Analysis

The calculation results of the HRV indices and subjective thermal sensation votes were presented as mean and standard deviation. Most of the HRV indices were not normally distributed when calculating each HRV index using Kubios Hrv3.3. The Mann-Whitney U test is a nonparametric test that does not require the distribution of raw data. Therefore, the Kruskal-Wallis test was used to analyze the significance of each HRV index under different temperature conditions. The Spearman correlation coefficient, which can be used to measure the strength of the link between two variables, is also a statistical parameter of a nonparametric nature. Here, bivariate Spearman correlation analyses between various HRV indices and subjects’ thermal sensation votes were explored under different temperature conditions.

## 3. Results

**Figure 2:**
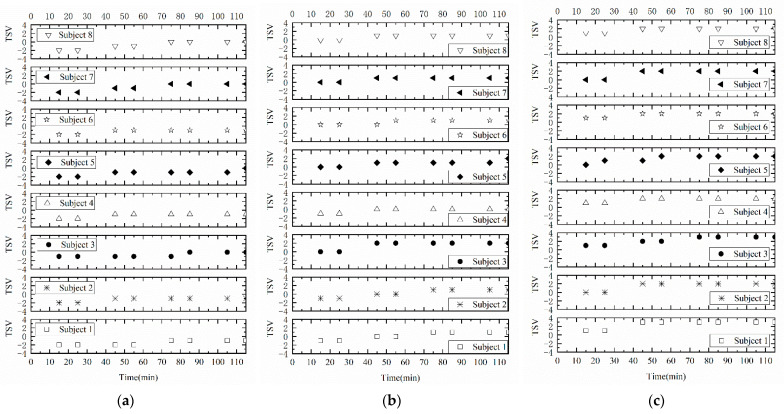
The thermal sensation votes of the subjects: (**a**) 22 °C/90%; (**b**) 26 °C/90%; (**c**) 30 °C/90%.

**Figure 3:**
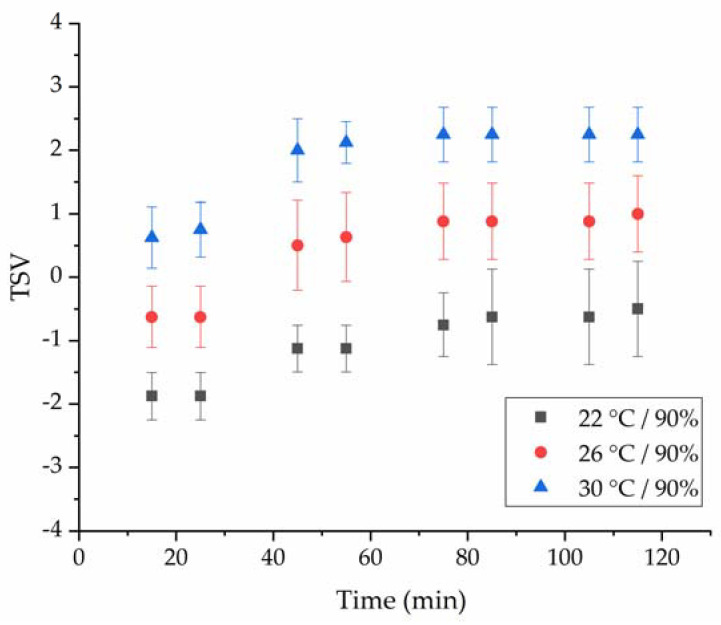
The subjects’ mean thermal sensation vote over the course of the experiment.

The ECG and subjective thermal sensation votes of the subjects were obtained in this experiment. In the three kinds of thermal environments, the subjective thermal sensation votes of the subjects were between −1.88 and 2.38, as shown in Figure 2 and Figure 3. Various HRV indices were calculated, and they changed significantly in different temperature environments, as showed in Table 4, Table 5, Table 6, Table 7, Table 8, Table 9, Table 10, Table 11, Table 12 and Table 13. Except for the LF, TP and LF/HF, there were significant differences in the other HRV indices in different temperature environments (Mann-Whitney U test, *p* < 0.05). The VLF, pNN20, R-R and SampEN interval had strong negative correlation with the thermal sensation of working people, and the correlation coefficient of the Spearman test was less than −0.717. From Figure 2a–c, it can be seen that there were some differences in the thermal sensation votes (TSVs) of the subjects during different running stages of simulated heavy labor. Different subjects reported different levels of thermal sensation to the same temperature environment. However overall the subjects felt slightly warm at 26 °C/90% environment, hot or very hot at 30 °C/90%, and the TSVs of the subjects at 22 °C/90% environment were distributed in the range of cold, slightly cold to neutral.

In the same thermal environment, there was a certain fluctuation in each HRV index as the subjects ran a course. The R-R interval showed the least fluctuation, and several indices including the LF, HF, TP and LF/HF, fluctuated more during the running progress. The relatively large fluctuations in these HRV indices may have affected the significant differences between different temperature environments at some measurement moment. There was no significant difference of the LF, TP and LF/HF between three different temperature environments (*p* > 0.05). All of the other HRV indices were significantly different (*p* < 0.05) between the hot environments and neutral environment (26 °C/90%), there was no significant difference between the cold environment (22 °C/90%) and neutral environment (26 °C/90%) For the SampEN, we found that it was the largest in the neutral environment (26 °C/90%), while it decreased in the cold and hot uncomfortable environment. This is different from the experimental results of Kizito et al. [18].

Under different temperature conditions, during the experimental period of sitting to run, the correlation between each index and the subjective thermal sensation votes of the subjects showed great differences, as shown in Table 14. Among them, six indices, SDNN, RMSSD, LF, HF, TP and LF/HF showed insignificant correlations (*p* > 0.05) or not strong enough correlations (ρ < 0.7 or ρ > −0.7) with thermal sensation in a constant temperature environment. Surprisingly, the LF/HF did not respond well to the thermal sensation of the subjects during running in different thermal environments. It was also considered as a good indicator of human thermal sensation in some studies on sitting or resting human body [21,25,26]. Strong negative correlations were found between the R-R interval, VLF, pNN20 and SampEN indices, and subjective thermal sensation. Their Spearman correlation coefficients satisfied ρ < −0.700, and were statistically significant (*p* < 0.05). Among them, the strongest correlation with thermal sensation was the SampEN, with correlation coefficients ρ = −0.860 (*p* = 0.006), and −0.867 (*p* = 0.005), −0.913 (*p* = 0.002) in the cold, neutral, and hot environments, respectively. The spectral-domain indices, including the LF, HF, and TP, showed strong negative correlations with subjective thermal sensation in both the neutral and hot environments, which were also statistically significant (*p* < 0.05). However, neither a strong correlation nor statistical significance existed with subjective thermal sensation in the cold environments.

## 4. Discussion

Regarding the research and evaluation of the thermal comfort of the operator body in the mining environment, the most widely used at present is the WBGT index. However it cannot reflect the physiological and psychological conditions of the human body. Another thermal stress model, PHS, commonly used in mining production management, can only make predictions on the physiological parameters of the human body, not human psychology. Heart rate variability (HRV) has been considered a good biomarker of thermal comfort in some previous studies. The purpose of this study was to experimentally investigate whether HRV can express the thermal comfort condition of workers in mining environments. The experimental studies were carried out under three kinds of environments representative of cold, neutral and hot in mines. In general, most subjects reported feeling cold, slightly cold or neutral in a cold environment, slightly warm in a neutral environment, and hot or very hot in a hot environment.

However, in a constant temperature thermal environment, there were differences in the level of thermal sensation among different subjects. This may be caused by bias at of voting, but some researchers believe that different people have different thermal preferences [33,34]. This may also be a consequence of differences in the perception of thermal activity between subjects. In all cases, however, no subject reported feeling hot in the cold environment or cold in the hot environment. That is, the subjects felt cold in the cold environment and felt hot in the hot environment. Therefore, the thermal sensation vote of the subjects in 3 thermal environments was objective.

It was found in the experiment that the LF, HF, and TP of the subjects all decreased during the running stage under a constant temperature environment. This reflects the transition from vagal to sympathetic dominance of humans after starting with running [35,36,37,38]. These indices all decreased in the cold or hot uncomfortable environment, reflecting the stimuli of the external thermal environment for the human body, and the autonomic nervous system was regulated. Surprisingly, the LF, TP and LF/HF indices were significantly different (*p* < 0.05) between the neutral and hot environments but not between the cold and neutral environments (*p* > 0.05). As seen from the thermal sensation votes of subjects, the subjective perception of the human body when running in a cold environment was changed from cold, slightly cold, to neutral. At the beginning of running, vasoconstriction was induced by hypothalamic sympathetic activity under the stimulation of a cold environment [20]. This decreases blood flow to human skin, and the heat dissipation of skin is reduced. When the human body temperature increased after undergoing a running period, a relatively stable heat balance between the human body and the outside world was established. The sympathetic activity was reduced at this time. We observed that the subjects sweated later in the running stage. This indicated that the human body was in a physiological thermal state at this time. The activity of sweat glands indicated that the sympathetic nerves were active again, at which point the sympathetic nerve that was the cerebral cortex predominated [20]. The fluctuating situation of the LF under a cold environment reflects the above changing process of the sympathetic nerve. The fluctuation of the LF caused a dramatic change in the TP and LF/HF simultaneously. However, the thermal sensation vote of the subjects reacted to their psychological state of comfort. It may be that the externally cold environment meets people’s psychological expectations. This also resulted in the LF, HF, and TP being uncorrelated with the subjective thermal sensation in the cold environment (22 °C/90%). However, the subjects were always in a hot condition physiologically when running in a neutral or hot environment. The activity of the sympathetic nerve was relatively stable, and the fluctuation of the LF was relatively small. In general, the LF, HF and TP truly reflect the physiological activities of the human body in different thermal environments. In some thermal environments, they are not correlated to the human thermal sensation, which indicates the possible inconsistency between human psychological sensation and physiological sense.

The LF/HF fluctuated to a greater extent in the three thermal environments, and there were no significant differences between them. There were no were there significant correlations between them and the subjective thermal sensation votes of subjects. This is because, in addition to the autonomic nervous system, the mechanical or neural coupling among the heart, motor system and respiratory system also affects the HRV during strenuous exercise [36]. The large increase in the respiratory rate and the failure of autonomic control of the cardiac vagus nerve cause HF to remain active [35,37]. Notably, the LF/HF is a response of sympathetic and parasympathetic balance in humans. It has also been considered a good indicator of the thermal sensation of the human body [21,25,26]. Nevertheless, their conclusions were obtained for sedentary or resting human subjects. In this experiment, for the human body of simulated labor, the LF/HF did not respond well to the stimulation of the human body by different thermal environments. Therefore, LF/HF index, which can express human thermal sensation at low metabolic rate, was not necessarily able to express human thermal sensation at a high metabolic rate.

The R-R interval was the most stable during the entire run under an environment of constant temperature. The R-R interval was largest in the comfortable environment, while it decreased smaller in the cold and hot uncomfortable environments. Significant changes (*p* < 0.01) in the R-R interval were observed among subjects exposed to different temperature environments. The R-R interval of humans was intrinsically influenced by ambient temperature and skin vasomotion [24]. It was also found in this experiment to have a strong correlation with human subjective thermal sensation. The time-domain index pNN20 obtained according to the R-R interval statistics was similar to the case of the R-R interval. It was maximal in comfortable environments and decreased in both cold and hot uncomfortable environments. This reflected that the functional level of the vagus nerve was suppressed in an uncomfortable environment. The pNN20 also has a strong correlation with the subjective thermal sensation of the human body.

The VLF was also of great interest for our study. Its corresponding signal frequency is 0.00–0.04 Hz, which results from spectral component analysis of the variability of successive R-R intervals. It well reflects the role of the vagal system on the HRV of the human body. The VLF is also thought to be associated with thermoregulatory activity in humans [28]. It was greatest in cold environments and decreased in hot environments. In this experiment, the VLF was the largest in the cold environment (22 °C/90%) and decreased in the neutral and hot environment. This is because the subjects were engaged in high-intensity exercise in the cold environment, the heat dissipation effect through the body surface was relatively strong, and the heat regulation activity in the body was not intense. It can also be seen from the heat feeling poll that with the running, the subjects’ heat feeling was very close to the heat neutral. When people were engaged in heavy labor in a hot environment, the heat dissipation of the body relies mainly on evaporative heat dissipation. Although the subject sweated profusely and drenching was observed in the hot environment of this experiment, the near saturated humidity of the air made evaporative heat dissipation of the body surface difficult. At this time, the VLF became very small. A very strong negative correlation between the VLF and subjective thermal sensation of the subjects was found in the experiments. Therefore, it can serve as a good indicator of the thermal sensation of the worker. Kizito et al. [18] also considered it a good indicator of thermal sensation when studying the HRV of sedentary humans.

The SampEN was an important method to quantify signal predictability. In this experiment, it was found that under three temperature conditions, the SampEN of the subjects in the running stage was lower than that of the set in the stage. This result is consistent with the experimental results of Shi B. et al. [38,39]. Among them, in the experiment of Shi B. et al., the walking speed of the subjects is 5.0 km/h, which is very close to the running speed in this test. The ambient temperature was controlled at 23 °C. The experimental condition is close to the cold environment. In this experiment, the SampEN was the largest in the neutral environment (26 °C/90%), but it decreased in the cold and hot uncomfortable environment. However, in Kizito et al.’s experiments, it seems that sample entropy was the highest in non-thermal comfort environment (hot and cold) and the lowest in comfortable environment (neutral). This is different from the experimental results in this paper. Whether this is caused by the differences in sport, clothing and wind speed remains to be proved by further experimental research. Nevertheless, in this experiment, the SampEN had the strongest correlation with the subjects’ thermal sensation votes.

Thermal comfort is related not only to human physiology but also to human psychology. There may be differences in subjects’ subjective thermal sensation voting, which is related to physiological differences, thermal perception, and thermal preference in humans. Heat stress and emotion may also have effects on subjects during prolonged running. We kept the subjects emotionally stable during the experiment by working efforts such as playing music. This, however, also cannot completely rule out possible inaccuracies in subjects’ thermal sensation voting and the HRV. In addition, for people who were exercising, breathing has a greater impact on the HF. There was no direct measurement of respiratory rate in this test. This is also a limitation of this study. The SampEN of HRV was calculated in this study, but the SampEN was only analyzed on a time scale, and the results may deviate from the actual situation, especially for the working people. In the later research, we will use the multi-scale entropy method to study the relationship between HRV and thermal sensation. It can better consider the different time scales that may exist in the time series.

## 5. Conclusions

In this experiment, three mine environments, 22 °C/90%, 26 °C/90% and 30 °C/90%, which represent cold, neutral and hot environments, respectively, were created through an artificial environmental chamber. Subjects were recruited to simulate heavy labor by running, and the HRV time-domain and spectral-domain indices were obtained by measuring ECG. The variation in each HRV indices of the human body and their correlation with subjective thermal sensation were studied. The results showed that:

In the neutral and hot environment, except for the LF, TP and LF/HF, there were significant differences in each index. However, there was no significant difference between the cold and neutral environments. Among them, the VLF indices were the largest in cold environments and decreased as ambient temperature increased. Other indices were all the highest in neutral environments and were reduced in cold and hot uncomfortable environments. In the case of a large difference in metabolic rate, the correlation between LF/HF and the human thermal sensation was not consistent.

The cardiac sympathetic nerves of workers in an uncomfortable environment were active, and the parasympathetic nerves were inhibited. The sympathetic activity was reduced and the parasympathetic was active in a thermally comfortable environment.

In different temperature environments, R-R interval, VLF, pNN20 and SampEN showed strong correlation with the subjective thermal sensation of human body, which can express thermal status of the people from sitting to work. They may serve as indicators of the thermal comfort for workers in underground mining environments.

Based on these indices, it is possible to investigate further and establish a thermal comfort evaluation system oriented to workers in mining and other industries. The application of this evaluation system of thermal comfort in the industry will be beneficial to grasp the thermal comfort of workers, which is used to protect the health of workers, and can manage production more reasonably.

## Figures and Tables

**Table 1:** Experimental Procedure.

Session No.	Duration (Min)	Stages	Events
1	15	Preparation	Install the instruments, measure the initial parameters
2	25	sitting	Vote at 15 and 25 min
3	5	Rest	
4	25	Running	Simulated labor, vote at 15 and 25 min
5	5	Rest	
6	25	Running	Simulated labor, vote at 15 and 25 min
7	5	Rest	
8	25	Running	Simulated labor, vote at 15 and 25 min
9	5	Rest	

**Table 2:** Climate Chamber Thermal Environment Settings.

Types	Air Temperature (°C)	Radiant Temperature (°C)	Humidity (%RH)	Air Speed (m/s)	PMV Index *
Cool	22	22	90	1.5	−1.05
Neutral	26	26	90	1.5	0.45
Hot	30	30	90	1.5	2.01

* Calculated as a human wearing a neat cotton coverall sitting still in that environment.

**Table 3:** Short Description of the Selected HRV Indices.

Time-Domain HRV Indice	Short Description
Mean RR	Average of the RR intervals
SDNN	Mean of the standard deviations of RR intervals
RMSSD	Square root of the mean squared differences between successive RR intervals
pNN20Spectral HRV indice	NN20 divided by the total number of RR intervals
LF	Spectral power in low range frequencies (0.04–0.15 Hz)
HF	Spectral power in high range frequencies (0.15 Hz)
VLF	Spectral power in very low range frequencies (0.000–0.04 Hz)
TP	Total spectral power (0–0.4 Hz)
LF/HF	Ratio between LF and HF band powers
SampEN	Sample entropy—a measure of complexity

Note: The above indices were based on the 5-min segments.

**Table 4:** The Mean RR during the Tests.

Environment Condition	22 °C/90%	26 °C/90%	30 °C/90%
Sitting	(768.67 ± 55.72)	(840.13 ± 75.80)	(802.71 ± 98.41)
	(768.83 ± 52.63)	(849.50 ± 84.39)	(802.00 ± 92.34)
Running	(537.50 ± 71.44)	(595.00 ± 68.95)	(516.57 ± 68.87)
	(539.50 ± 64.39)	(590.75 ± 71.52)	(497.43 ± 71.47)
Running	(541.00 ± 53.70)	(590.75 ± 61.12)	(498.86 ± 77.95)
	(534.67 ± 66.28)	(534.67 ± 66.28)	(482.14 ± 69.30)
Running	(542.83 ± 57.98)	(591.88 ± 74.28)	(496.00 ± 65.02)
	(530.83 ± 58.10)	(583.25 ± 63.94)	(475.00 ± 58.44)

Note: The data in the table are the mean and standard deviation of the 10th to 15th min and the 20th to 25th min of the sitting or running stage.

**Table 5:** The SDNN during the Tests.

Environment Condition	22 °C/90%	26 °C/90%	30 °C/90% **
Sitting	(40.58 ± 14.19)	(45.74 ± 19.71)	(38.67 ± 14.07)
	(44.72 ± 18.90)	(50.53 ± 19.03)	(37.77 ± 10.61)
Running	(11.40 ± 6.67)	(13.95 ± 6.34)	(8.39 ± 4.51)
	(10.60 ± 5.31)	(13.79 ± 5.76)	(7.36 ± 3.70)
Running	(10.38 ± 4.93)	(14.41 ± 5.93)	(7.04 ± 3.97)
	(11.27 ± 5.54)	(15.08 ± 8.28)	(6.83 ± 4.65)
Running	(12.67 ± 6.01)	(13.80 ± 6.67)	(6.89 ± 3.16)
	(12.27 ± 6.58)	(12.61 ± 4.86)	(6.71 ± 2.17)

Note: The data in the table are the mean and standard deviation of the 10th to 15th min and the 20th to 25th min of the sitting or running stage. ** Indicates a significant difference between the 30 °C/90% environment and the 26 °C/90% environment.

**Table 6:** The RMSSD during the Tests.

Environment Condition	22 °C/90%	26 °C/90%	30 °C/90% **
Sitting	(38.27 ± 17.52)	(51.39 ± 19.25)	(35.56 ± 17.13)
	(43.38 ± 24.13)	(55.08 ± 24.07)	(36.71 ± 16.78)
Running	(10.12 ± 6.64)	(13.01 ± 6.58)	(7.27 ± 4.09)
	(8.82 ± 5.12)	(13.89 ± 6.42)	(6.87 ± 3.78)
Running	(7.87 ± 3.62)	(14.99 ± 7.84)	(6.53 ± 3.39)
	(8.95 ± 5.54)	(15.41 ± 9.94)	(6.34 ± 3.08)
Running	(9.13 ± 4.99)	(13.15 ± 7.13)	(6.44 ± 2.66)
	(8.28 ± 5.19)	(13.61 ± 7.97)	(5.70 ± 1.92)

Note: The data in the table are the mean and standard deviation of the 10th to 15th min and the 20th to 25th min of the sitting or running stage. ** Indicates a significant difference between the 30 °C/90% environment and the 26 °C/90% environment.

**Table 7:** The pNN20 During the Tests.

Environment Condition	22 °C/90%	26 °C/90%	30 °C/90% **
Sitting	(53.42 ± 14.14)	(63.14 ± 13.13)	(48.31 ± 20.58)
	(56.39 ± 14.96)	(63.22 ± 13.47)	(49.56 ± 15.98)
Running	(8.30 ± 13.33)	(11.65 ± 15.41)	(2.95 ± 5.98)
	(6.03 ± 10.80)	(14.21 ± 15.71)	(3.24 ± 7.20)
Running	(7.32 ± 12.70)	(13.00 ± 11.70)	(3.02 ± 6.31)
	(6.69 ± 11.70)	(12.41 ± 11.83)	(2.15 ± 4.78)
Running	(6.41 ± 10.70)	(10.53 ± 11.58)	(2.48 ± 4.55)
	(5.44 ± 9.80)	(7.14 ± 7.10)	(2.20 ± 5.03)

Note: The data in the table are the mean and standard deviation of the 10th to 15th min and the 20th to 25th min of the sitting or running stage. ** Indicates a significant difference between the 30 °C/90% environment and the 26 °C/90% environment.

**Table 8:** The LF during the Tests.

Environment Condition	22 °C/90%	26 °C/90%	30 °C/90%
Sitting	(941.75 ± 477.21)	(1213.25 ± 89.03)	(968.01 ± 669.61)
	(976.13 ± 742.36)	(1217.50 ± 957.65)	(988.13 ± 590.51)
Running	(97.38 ± 104.83)	(136.10 ± 116.64)	(45.51 ± 38.51)
	(87.25 ± 98.50)	(140.51 ± 109.48)	(43.00 ± 39.05)
Running	(78.88 ± 73.56)	(112.51 ± 89.25)	(39.88 ± 37.89)
	(86.63 ± 90.95)	(120.25 ± 113.95)	(34.25 ± 50.14)
Running	(89.25 ± 89.27)	(100.25 ± 78.79)	(41.88 ± 33.65)
	(85.88 ± 60.87)	(98.51 ± 61.97)	(29.25 ± 18.00)

Note: The data in the table are the mean and standard deviation of the 10th to 15th min and the 20th to 25th min of the sitting or running stage.

**Table 9:** The HF during the Tests.

Environment Condition	22 °C/90%	26 °C/90%	30 °C/90% **
Sitting	(798.01 ± 567.93)	(1073.82 ± 1027.14)	(611.58 ± 445.10)
	(802.43 ± 983.92)	(1043.10 ± 922.82)	(595.38 ± 506.37)
Running	(11.60 ± 7.39)	(45.37 ± 73.92)	(7.75 ± 6.51)
	(14.37 ± 12.56)	(34.17 ± 36.82)	(8.88 ± 9.68)
Running	(14.12 ± 8.74)	(39.51 ± 43.45)	(7.50 ± 7.97)
	(15.41 ± 12.00)	(38.22 ± 45.61)	(7.25 ± 8.10)
Running	(13.60 ± 8.24)	(21.30 ± 23.42)	(8.38 ± 9.02)
	(16.29 ± 12.33)	(26.17 ± 25.13)	(5.88 ± 5.73)

Note: The data in the table are the mean and standard deviation of the 10th to 15th min and the 20th to 25th min of the sitting or running stage. ** Indicates a significant difference between the 30 °C/90% environment and the 26 °C/90% environment.

**Table 10:** The VLF during the Tests.

Environment Condition	22 °C/90%	26 °C/90%	30 °C/90% **
Sitting	(142.50 ± 65.70)	(124.25 ± 69.01)	(109.40 ± 52.60)
	(147.38 ± 80.40)	(117.51 ± 49.35)	(103.75 ± 59.24)
Running	(24.88 ± 34.29)	(19.88 ± 12.83)	(11.50 ± 11.78)
	(19.63 ± 26.25)	(15.00 ± 11.37)	(10.25 ± 6.12)
Running	(22.63 ± 22.52)	(17.13 ± 8.95)	(5.88 ± 3.33)
	(20.38 ± 19.72)	(15.13 ± 12.32)	(5.25 ± 5.02)
Running	(20.61 ± 13.65)	(14.25 ± 8.33)	(8.63 ± 6.02)
	(21.94 ± 30.29)	(17.25 ± 9.26)	(8.00 ± 6.08)

Note: The data in the table are the mean and standard deviation of the 10th to 15th min and the 20th to 25th min of the sitting or running stage. ** Indicates a significant difference between the 30 °C/90% environment and the 26 °C/90% environment.

**Table 11:** The TP during the Tests.

Environment Condition	22 °C/90%	26 °C/90%	30 °C/90%
Sitting	(1797.67 ± 1130.51)	(2459.63 ± 2068.06)	(1627.10 ± 1159.99)
	(2046.00 ± 1974.17)	(2395.00 ± 2097.14)	(1646.25 ± 884.99)
Running	(136.83 ± 166.65)	(157.25 ± 174.32)	(63.75 ± 55.12)
	(120.33 ± 154.86)	(141.63 ± 123.94)	(60.25 ± 50.00)
Running	(112.83 ± 116.79)	(117.10 ± 97.81)	(52.25 ± 44.59)
	(123.67.38 ± 139.36)	(128.00 ± 151.82)	(45.00 ± 57.86)
Running	(204.33 ± 227.17)	(110.01 ± 102.84)	(57.13 ± 42.32)
	(173.67 ± 181.96)	(98.75 ± 58.37)	(42.13 ± 23.50)

Note: The data in the table are the mean and standard deviation of the 10th to 15th min and the 20th to 25th min of the sitting or running stage.

**Table 12:** The LF/HF during the Tests.

Environment Condition	22 °C/90%	26 °C/90%	30 °C/90%
Sitting	(1.15 ± 1.28)	(4.91 ± 3.04)	(2.23 ± 1.68)
	(1.84 ± 0.82)	(5.50 ± 2.18)	(1.88 ± 1.23)
Running	(8.05 ± 4.22)	(4.90 ± 2.93)	(7.90 ± 2.90)
	(7.50 ± 4.04)	(3.33 ± 1.37)	(7.03 ± 3.75)
Running	(6.34 ± 5.44)	(5.12 ± 2.41)	(6.96 ± 5.75)
	(5.92 ± 2.08)	(6.58 ± 4.17)	(4.85 ± 2.52)
Running	(6.56 ± 4.14)	(1.38 ± 0.70)	(6.80 ± 3.59)
	(5.27 ± 3.03)	(1.13 ± 0.47)	(9.38 ± 10.99)

Note: The data in the table are the mean and standard deviation of the 10th to 15th min and the 20th to 25th minute of the sitting or running stage.

**Table 13:** The SampEN during the Tests.

Environment Condition	22 °C/90%	26 °C/90%	30 °C/90% **
Sitting	(1.84 ± 0.09)	(1.84 ± 0.29)	(1.79 ± 0.17)
	(1.79 ± 0.17)	(1.94 ± 0.12)	(1.85 ± 0.50)
Running	(1.59 ± 0.21)	(1.75 ± 0.19)	(1.51 ± 0.35)
	(1.62 ± 0.24)	(1.78 ± 0.21)	(1.41 ± 0.42)
Running	(1.54 ± 0.17)	(1.75 ± 0.24)	(1.37 ± 0.41)
	(1.60 ± 0.18)	(1.73 ± 0.20)	(1.30 ± 0.31)
Running	(1.55 ± 0.22)	(1.69 ± 0.17)	(1.39 ± 0.41)
	(1.48 ± 0.22)	(1.72 ± 0.29)	(1.32 ± 0.28)

Note: The data in the table are the mean and standard deviation of the 10th to 15th min and the 20th to 25th min of the sitting or running stage. ** Indicates a significant difference between the 30 °C/90% environment and the 26 °C/90% environment.

**Table 14:** Bivariate Correlation between the HRV Indices and TSV.

HRV Indices	TSV		
	22 °C	26 °C	30 °C
Mean RR	(−0.727, 0.041)	(−0.902, 0.002)	(−0.875, 0.004)
SDNN	(−0.364, 0.376)	(−0.675, 0.066)	(−0.939, 0.001)
RMSSD	(−0.691, 0.058)	(−0.454, 0.258)	(−0.913, 0.002)
pNN20	(−0.800, 0.017)	(−0.798, 0.018)	(−0.799, 0.017)
LF	(−0.364, 0.376)	(−0.970, 0.001)	(−0.786, 0.021)
HF	(−0.291, 0.484)	(−0.971, 0.000)	(−0.799, 0.017)
VLF	(−0.717, 0.045)	(−0.778, 0.023)	(−0.939, 0.001)
TP	(−0.364, 0.376)	(−0.920, 0.000)	(−0.786, 0.021)
LF/HF	(0.582, 0.130)	(0.356, 0.387)	(0.558, 0.151)
SampEN	(−0.860, 0.006)	(−0.867, 0.005)	(−0.913, 0.002)

Note: Correlation analysis is based on the process from sitting to running. Values are expressed as (ρ, *p*).

## Data Availability

The data presented in this study are available from the corresponding author on reasonable request. The data are not publicly available due to restrictions eg privacy or ethical.
